# Isolated Meniscus Tears in Adolescent Patients Treated with Platelet-Rich Plasma Intra-articular Injections: 3-Month Clinical Outcome

**DOI:** 10.1155/2020/8282460

**Published:** 2020-05-21

**Authors:** Mihai Bogdan Popescu, Madalina Carp, Iulia Tevanov, Catalin Alexandru Nahoi, Madalina Andreea Stratila, Oana Mihaela Haram, Alexandru Ulici

**Affiliations:** Department of Pediatric Orthopedic Surgery, Emergency Hospital for Children “Grigore Alexandrescu”, Bucharest, Romania

## Abstract

**Objectives:**

Meniscus repair is a challenge for a practitioner, as an injured meniscus can lead to osteoarthritic joint changes with a greatly disabling outcome. Platelet-rich plasma has been regarded as a promising therapy to help induce healing. The purpose of the study is to clinically assess the effectiveness of PRP treatment in adolescents with meniscal lesions.

**Methods:**

This retrospective study analyzed 30 patients with meniscal tears, aged 12 to 17 years, who had documented MRI meniscal lesion and persistent knee pain. In order to evaluate the outcome, the Lysholm knee scoring scale and numerical rating scale were used before injection and 3 months after treatment.

**Results:**

Patients had a mean age of 13.93 years, 70% girls and 30% boys. The most affected was the medial meniscus. The mean value before injection on the numerical rating scale (NRS) of pain was 7.73, after the treatment being of 2.0. After treatment, 76.7% of the patients had “excellent” and “good” outcomes, while before injection, just 3% of the patients had a “good” score.

**Conclusions:**

Platelet-rich plasma treatment can be effective in improving the clinical outcomes of adolescent patients with meniscus tears, for whom conservative management and physical therapy have failed to achieve pain relief.

## 1. Introduction

Adolescent participation in athletics increased lately and, with it, an inflation of sport-related injuries such as meniscal tears, leading to possible unfavorable effects on the growing skeleton in the long term [1].

The importance of the menisci is highlighted after complete or partial meniscectomies that may lead to osteoarthritic and degenerative changes, as menisci are structures that are critical to shock absorption, load sharing, and stability within the joint [2, 3]. From birth, the vascularized area of the meniscus recedes by the age of 10, when only the peripheral 10-30% is vascularized [4]. The good vascularization of the meniscus in young individuals is responsible for its healing capacity [5]. In lesions occurring in the avascular zone, the healing process is mainly based on the self-repair capacity of the meniscal tissue [6]. Based on MRI, Reicher et al. divided meniscal lesions into 4 grades [7]. In a grade 1 lesion, there is mucoid degeneration without signs of rupture. Physical therapy is the main treatment approach for this type of injury [8]. For grade 3 lesions, there are complete meniscal tears with an interruption of the meniscal surface; meniscal repair by surgical intervention is the treatment of choice; for older or low-demand patients, partial meniscectomy is preferred [9]. Grade 2 lesions can be considered a prestage meniscal rupture. It is mainly an intrasubstance defect without surface disruption. Intrasubstance meniscal lesions can lead to a reduced physical activity or a complete rupture along with persistent pain. This is the main reason why conservative treatment is often not satisfactory [9, 10]. Meniscal repair should be considered in young patients when conservative treatment fails [11]. When physical therapy is not satisfactory, biological factors can be used to promote biological response [11].

Extra-articular ligaments heal by the following precise steps: after bleeding, a fibrin-platelet clot forms at the lesion site and fills the gap between the tissue ends, forming a scaffold for the surrounding cells [12]. In intra-articular lesions, synoviocytes stimulate the production of a urokinase plasminogen activator which converts the inactive plasminogen into plasmin which quickly degrades fibrin [13]. This leads to an unstable clot; the loss of the scaffold is one of the reasons why intra-articular tissues fail to heal [14]. Considering these aspects, intra-articular tissues such as meniscus or anterior cruciate ligament (ACL) have poor healing capacity. Medical research has become more oriented towards finding methods to enhance the biological response of these tissues [15]. Platelet-rich plasma (PRP) is an autologous blood product that contains a very high concentration of platelets. It contains a multitude of growth factors released by the platelets: transforming growth factor *β*1 (TGF-*β*1), fibroblast growth factor-2 (FGF-2), insulin-like growth factor 1 (IGF-1), epidermal growth factor, platelet-derived growth factor (PDGF), and vascular endothelial growth factor (VEGF). The regenerative effects of these growth factors, such as cell proliferation, migration, angiogenesis, and extracellular matrix production, had been demonstrated both in in vivo and in vitro models [16–18]. In the knee, besides the treatment of ligamentous and meniscal injuries, PRP has also been used successfully to treat articular cartilage pathology [19].

The controversy about the effectiveness of biologics in orthopedics led to the need for standardizing the outcome measurements. Clinical outcome scores have been extensively used to evaluate patients after a vast array of procedures from cartilage restoration to ligament reconstructions [20].

In the revised Lysholm scale, scores are categorized as excellent (95–100), good (84–94), fair (65–83), and poor (<64) [21]. Pain is an important complaint of patients with knee injuries, thus making pain evaluation after knee surgery a crucial aspect of the outcome assessment. The numerical rating scale (NRS) consists of asking the patient to rate their perceived level of pain intensity on a scale from 0 to 10. It is possible to be administered verbally, and it can even be used in telephone interviews [22]. NRS have been proven to have good compliance and have shown high correlations with other pain assessment tools [23].

The purpose of the study is to clinically assess the effectiveness of PRP treatment in adolescents with meniscal lesions.

## 2. Materials and Methods

A retrospective study was conducted on 30 patients with meniscal tears that were admitted and treated between December 2015 and December 2018. The study was conducted with the approval of the hospital's ethics committee. The inclusion criteria were adolescent patients, age ranging from 12 to 17 years, patients who had documented MRI meniscal lesion grade II according to Reicher et al. [7], and persistent knee pain after at least one month of medical treatment (rest, physiotherapy, and anti-inflammatory drugs). Exclusion criteria were prior or associated knee injuries (anterior or posterior cruciate ligament injuries, osteochondritis dissecans, and collateral ligament injuries).

The same intra-articular injection technique and PRP system were used for each patient. After injection, all patients underwent the same recovery protocol; no cast or immobilization was applied. They were not allowed to bear full weight for one week and advised to limit their physical activities for one month. For the management of post injection pain, patients were not allowed to take NSAID. This recommendation was maintained for at least 6 months as NSAID interfere with platelet functions [24].

In order to evaluate the outcome, for each patient, the Lysholm knee scoring scale was conducted by the orthopedic surgeon before injection and 3 months after treatment. Also, before and after treatment, patients were asked to rate their pain on the numeric rating scale. Statistical analysis was performed with Statistical Package for the Social Sciences (SPSS) and Microsoft Office Excel using descriptive statistics and comparing means with the *t*-test.

## 3. Results

Patients had a mean age of 13.93 years, 70% girls and 30% boys. Regarding the site of injury, the right knee was the most affected (56.66%). The most frequent location for tears was in the medial meniscus (70%). The amount of time from the onset of pain until the PRP treatment ranged from 30 days to 770 days, with a mean of 235 days (Table 1).

The mean value on the NRS of pain was 7.73, before injection, after the treatment being of 2.0. After treatment, 76.7% of the patients had “excellent” and “good” outcomes, according to the Lysholm score, while before injection, just 3% of the patients had a “good” score. Comparing the mean scores for the pain scale and Lysholm score before and after injection, an improvement was observed in the mean values, which is statistically significant (Table 2).

There was no difference between boys and girls regarding the pre and post injection scores. The Lysholm score had a high standard deviation (SD), meaning that the values are widespread around the mean. Concerning the Lysholm score before injection, girls had a mean of 50.38 and males 57.1 (*p* = 0.4), while the score after the treatment was 89.81 for girls and 86 for boys. An improvement of the symptoms in females compared to male patients was registered, but not statistically significant (*p* = 0.64), mainly because the number of male patients included in the study was too small (Table 3).

Regarding the right/left knee, there were no differences in the evolution after the treatment. The medial meniscus was the most affected, and it had a better improvement rate after treatment based on the pain scale (*p* = 0.05). An improvement was also observed in the Lysholm score (96 vs. 85), but it was not statistically significant (Table 3).

**Figure 1:**
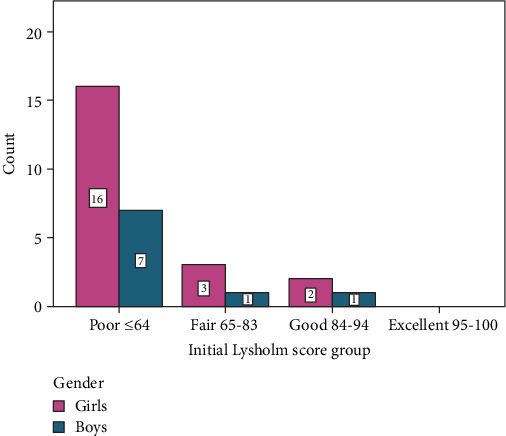
Initial Lysholm score.

**Figure 2:**
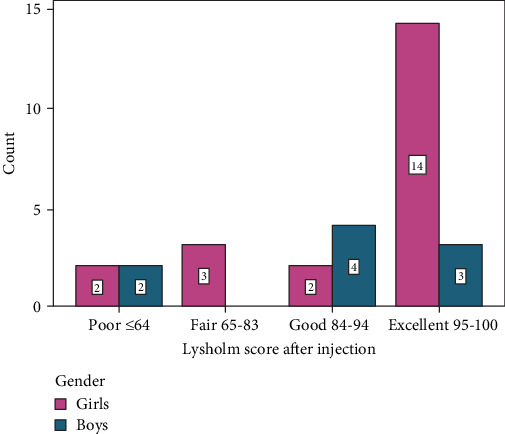
Lysholm score after the treatment.

There was no patient who had an initial “excellent” Lysholm score (Table 2, Figure 1). Before the treatment, 16 out of the 21 girls had a “poor” Lysholm score, while after the treatment, 14 had an “excellent” outcome (Figures 1 and 2).

## 4. Discussion

Meniscus repair, although being intensively studied, is still a challenge for the practitioner. Platelet-rich plasma is regarded as a promising therapy to help induce healing with good results, both in vivo and in vitro, but there are limited studies concerning the clinical effects of PRP on meniscal repair. Ishida et al. investigated the use of PRP for meniscal tissue healing in an animal trial conducted on rabbits and in vitro on monolayer meniscal cell cultures, concluding that it enhances the regenerating properties of the inner, avascular meniscus [25]. Another study that was performed on 35 patients who underwent arthroscopic meniscus repair, for 15 cases of PRP being used as an augmentation method, showed no appreciable functional difference [26].

Blanke et al., in their clinical study, revealed that intra-articular injections of PRP could be considered as a treatment option in patients with intrasubstance meniscal lesions, as they have the ability to achieve pain relief and stop progression on MRI [11]. A recent case report described the efficacy and favorable outcome of PRP treatment in a patient with grade 3 medial meniscus tear, during a 30-month follow-up [27]; in our study, we used a leukocyte-rich PRP that is associated with proinflammatory effects [28].

The demographic data of the patients included in our study, regarding the site of the lesion, was in accordance with most literature data, the medial meniscus being the most affected, even though there are studies which depict the lateral meniscus as being the most affected [29, 30]. In our study, girls were the most affected, probably because of the inclusion criteria and the small number of patients the study was conducted on; literature data shoes a higher incidence of meniscus lesions in boys [30, 31].

Even though male patients had a higher Lysholm score prior to injection, this did not translate into the final results, as the male patients had a lower score than female subjects. However, both the male and female patients had similar scores on the numerical rating scale of pain. At the 3-month follow-up, pain relief was achieved for the majority of patients, which was noticeable on both the numerical scale for pain and Lysholm score.

One main limitation of the study is the small number of cases. Another limitation is that pain is subjective, and the results of the numerical rating scale might be influenced by the variability in pain tolerance of each patient. Even though short-term results are encouraging, further studies with control groups and a long-term follow-up are necessary. In further studies, we could use post injection MRI to determine the lesion evolution.

At the three-month follow-up, the patients did not report any local side effects (swelling, redness, or infection) or worsening of their symptoms (increase in pain intensity, knee locking).

## 5. Conclusion

Platelet-rich plasma treatment can be effective in improving the clinical outcomes of adolescent patients with intramural meniscus tears, for whom conservative management with physical therapy has failed to achieve pain relief.

## Figures and Tables

**Table 1:** Demographic data.

Variable	Study group (*n* = 30)
Age (years)	13.93^∗^ ± 1.43^∗∗^ (range 12-17)
Sex, *n* (%)	
Female	21 (70)
Male	9 (30)
Affected knee, *n* (%)	
Left	13 (43.33)
Right	17 (56.66)
Side of tear, *n* (%)	
Medial meniscus	21 (70)
Lateral meniscus	9 (30)
Days from debut to treatment (days)	235^∗^ (range 30-770)

^∗^Mean, ^∗∗^±standard deviation.

**Table 2:** Outcome results.

Variable	Before injection	After injection
Numerical rating scale, mean ± standard deviation (*p*)	7.73 ± 1.5 (0.0)	2.0 ± 2.4 (0.0)
Lysholm score, mean ± standard deviation (*p*)	52.4 ± 19.79 (0.0)	88.67 ± 19 (0.0)
Poor, *n* (%)	23 (76.7)	4 (13.3)
Fair, *n* (%)	4 (13.3)	3 (10)
Good, *n* (%)	3 (10)	6 (20)
Excellent, *n* (%)	0 (0)	17 (56.7)

**Table 3:** Pain scale and Lysholm score—mean values before and after treatment.

	Pain scale before injection^∗^	*p*	Pain scale after injection^∗^	*p*	Lysholm score before injection^∗^	*p*	Lysholm score after injection ^∗^	*p*
Female	7.67	0.71	2	0.74	50.38	0.4	89.8	0.64
Male	7.89		2.33		57.11		86	
Right knee	7.42	0.2	1.42	0.29	55	0.7	91	0.9
Left knee	8.06		2.4		52.53		90	
Medial meniscus	7.33	0.3	0.78	0.05	60	0.14	96	0.15
Lateral meniscus	7.9		2.67		48		85	

^∗^Mean value.

## Data Availability

The authors confirm that the data supporting the findings of this study are available within the article. Supplementary materials that support the findings of this study are available on request from the corresponding author.
